# Two new *Dolichothele* Mello-Leitão, 1923 species from Brazil and Bolivia (Araneae, Theraphosidae)

**DOI:** 10.3897/zookeys.724.20680

**Published:** 2017-12-21

**Authors:** Irene Soliz Revollo, Pedro Ismael da Silva Júnior, Rogério Bertani

**Affiliations:** 1 UNESP, Universidade Estadual Paulista Júlio de Mesquita Filho, Instituto de Biociências, Letras e Ciências Exatas, Rua Cristóvão Colombo, 2265, 15054-000, São José do Rio Preto, São Paulo, Brazil; 2 Instituto Butantan, Laboratório de Ecologia e Evolução, Av. Vital Brazil, 1500, 05503-900, São Paulo, São Paulo, Brazil; 3 Instituto Butantan, Laboratório Especial de Toxinologia Aplicada, Av. Vital Brazil, 1500, 05503-900, São Paulo, São Paulo, Brazil

**Keywords:** Bolivia, cerrado, Ischnocolinae, tarantula, taxonomy

## Abstract

Two new species of *Dolichothele* Mello-Leitão, 1923 are described from Brazil and Bolivia, *D. mottai* **sp. n.** from Distrito Federal and the state of Goiás, Brazil, and *D. camargorum* **sp. n.** from the state of Rondônia, Brazil, and the La Paz region, Bolivia. Males of the two new species resemble *Dolichothele bolivianum* (Vol, 2001) in having a small subapical keel on the distal embolus and females in particular by the short spermatheca. *Dolichothele bolivianum* is redescribed, and its geographical distribution is herein restricted to Bolivia and the state of Mato Grosso in Brazil.

## Introduction

The genus *Dolichothele* Mello-Leitão, 1923 was described based on a single species, *Dolichothele exilis* Mello-Leitão, 1923, from the state of Paraíba, Brazil, and included in Barychelidae. Later, Bücherl et al. (1971) examined the female holotype and transferred it to Theraphosidae, Ischnocolinae. Raven (1985), in his mygalomorph revision, considered the genera *Dolichothele* and *Goniodontium* Mello-Leitão, 1923 junior synonyms of *Hapalotremus* Simon, 1903 (Theraphosidae, Theraphosinae), but he was not followed by Schmidt (2002), who restored the genus *Dolichothele* and considered it *incertae sedis*. Recently, Lucas and Indicatti (2015) examined the holotype of the type species, *D. exilis*, synonymized *Oligoxystre caatinga* Guadanucci, 2007 and *Goniodontium muticum* Mello-Leitão, 1923 with *D. exilis* Mello-Leitão, 1923 and considered *Dolichothele* a senior synonym of *Goniodontium* and *Oligoxystre* Vellard, 1924, making several new combinations: *D. auratum* (Vellard, 1924), *D. bolivianum* (Vol, 2001), *D. diamantinensis* (Bertani, Santos & Righi, 2009), *D. dominguense* (Guadanucci, 2007), *D. mineirum* (Guadanucci, 2011), *D. rufoniger* (Guadanucci, 2007), and *D. tucuruiense* (Guadanucci, 2007).

Most of the species presently considered in *Dolichothele* were described in the genus *Oligoxystre*, which was revised by Guadanucci (2007, 2011). This author recognized eight species distributed in Brazil and Bolivia, one of which, *D. bolivianum*, as having “a very wide distribution, from central Brazil to eastern Bolivia” (Guadanucci 2007: 10). Guadanucci (2007) observed variations in color between populations of *D. bolivianum* but considered that the examined specimens had the same genitalia morphology and, therefore, belonged to the same species. Morphologically re-analyzing part of the specimens studied by Guadanucci (2007) and with additional material, two new species closely related to *D. bolivianum* were found, which are herein described.

## Materials and methods

Specimens of the following institutions were examined: **DZUB**, Departamento de Zoologia da Universidade de Brasília, Brasília; **IBSP**, Instituto Butantan, São Paulo; **MHNNKM**, Museo de Historia Natural Noël Kempff Mercado, Santa Cruz de la Sierra; **MNRJ**, Museu Nacional da Universidade Federal do Rio de Janeiro, Rio de Janeiro; **MZUSP**, Museu de Zoologia da Universidade de São Paulo, São Paulo; **UFMG**, Coleções Taxonômicas da Universidade Federal de Minas Gerais, Belo Horizonte.

All measurements are in millimeters and were obtained from the right appendages, unless they were missing or regenerated. For measuring larger structures, such as carapace, abdomen and appendages, a Mitutoyo digital caliper was used with an error of 0.005 mm, rounded up to two significant decimals. Appendages were measured from the dorsal aspect. Image captures of the structures were made with a Leica M205C dissecting microscope, with a Leica LAS montage and a LAS 3D module with which small structures such as eyes were measured.

The position of spines on legs and palp followed the terminology of Petrunkevitch (1925) with the modifications of Bertani (2001). Abbreviations used were as follows:

**ALE** anterior lateral eye,

**AME** anterior median eye,

**ap** apical,

**d** dorsal,

**ITC** inferior tarsal claw,

**PLE** posterior lateral eye,

**PLS** posterior lateral spinneret,

**PME** posterior median eye,

**PMS** posterior median spinneret,

**p** prolateral,

**r** retrolateral,

**STC** superior tarsal claw,

**v** ventral.

Maps of species distributions were made with the program ArcGIS 10. Geographical coordinates were obtained from the labels when available (primary source, indicated by parentheses) or using Google Earth (secondary source, indicated by brackets).

## Taxonomy

### Dolichothele

Taxon classificationAnimaliaAraneaeTheraphosidae

Mello-Leitão, 1923

Dolichothele Mello-Leitão, 1923: 119 (Type species by original designation D. exilis Mello-Leitão, 1923, type in MNRJ, not examined); Lucas and Indicatti 2015: 205.
Oligoxystre Vellard, 1924: 151, pl. 10, f. 38 (Type species by original designation O. auratum Vellard, 1924, should be deposited at Instituto Vital Brazil, Niterói, lost); first synonymized by Lucas and Indicatti 2015: 205.
Pseudoligoxystre Vol, 2001: 4–6, f. 7 (type species Pseudoligoxystre bolivianum Vol, 2001, deposited at MHNNKM, examined); first synonymized by Guadanucci 2007.
Goniodontium Mello-Leitão, 1923: 126 (type species by original designation Goniodontium muticum Mello-Leitão, 1923, type in MNRJ, not examined); first synonymized by Lucas and Indicatti 2015: 205.

#### Diagnosis

(from Guadanucci 2011). Differs from other ischnocoline and resembles genus *Catumiri* Guadanucci, 2004 by the labium much wider than long, bearing a reduced number of cuspules (fewer than 10). It differs from *Catumiri* Guadanucci, 2004 by the undivided tarsal scopula on legs I–III and scopula on tarsi IV undivided but with a longitudinal band of setae, the metatarsus I having scopula ventrally for all its length, the well-developed retrolateral branch of the male tibial apophysis, the tarsal claws of males without teeth, and by the spermathecae with numerous lobules.

### Dolichothele bolivianum

Taxon classificationAnimaliaAraneaeTheraphosidae

(Vol, 2001)

Figs 1–3 , 4–8 , 25 , 28–29 , 36

Pseudoligoxystre bolivianus Vol, 2001: 3, f. 1–7.
Oligoxystre bolivianum ; Guadanucci 2007: 4, f. 1–12 (only f. 9).
Dolichothele bolivianum ; Lucas and Indicatti 2015: 207; World Spider Catalog 2017.

#### Type material.

Holotype male, BOLIVIA: *Santa Cruz*: Samaipata [18°7'S; 63°53'W], September 2000, J. M. Verdez & H. Simoens coll. (MHNNKM 003).

#### Other material.

**BOLIVIA**: *Santa Cruz*: Samaipata [18°10'S; 63°50'W], 1 male, 06 October 2004, D. Weinmann & A. Stirm coll. (MZUSP 26083); 1 male, 07 October 2004, D. Weinmann & A. Stirm coll. (MUZSP 26082); 1 female, 28 December 2015, I. S. Revollo & R. B. Huanto coll. (MHNNKM unnumbered); BRAZIL: *Mato Grosso*: Chapada dos Guimarães [15°27'S; 55°44'W], 1 male, 19 March 1992, D. Pinz coll. (IBSP 109495); 1 male, February 1991, S. M. Lucas coll. (IBSP 109494); 1 female, 2000, equipe de resgate de fauna coll. (IBSP 109040); Cuiabá [15°36'S; 56°05'W], 1 male, January 1991, D. M. de Paula coll. (IBSP109496); Poconé [16°16'S; 56°37'W], 1 female (IBSP 109502).

#### Differential diagnosis.

**Figures 1–3.**
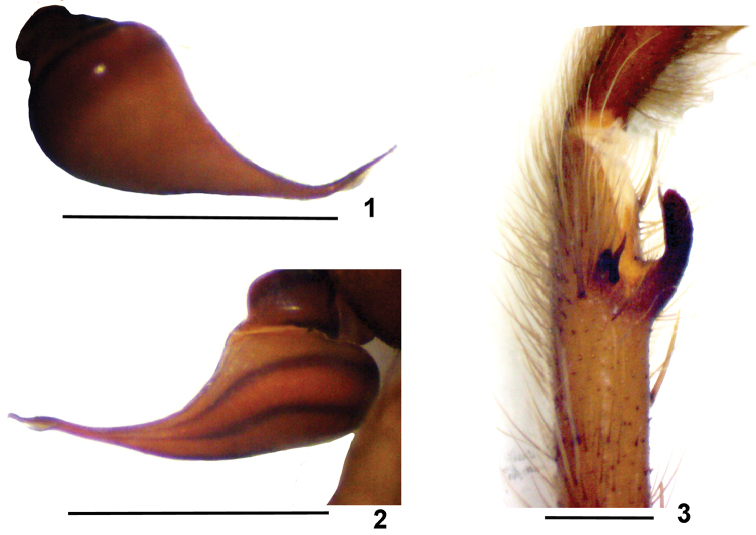
*Dolichothele bolivianum*. Holotype male. **1–2** right male palpal bulb **1** retrolateral view **2** prolateral view **3** left leg I tibial apophysis, prolateral view. Scale bar: 1 mm.

**Figures 4–8.**
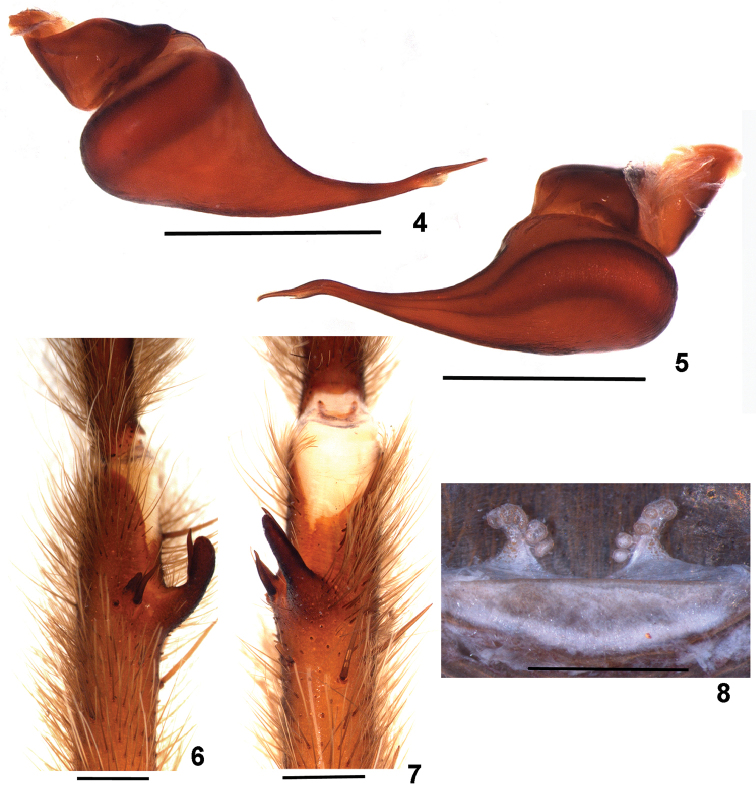
*Dolichothele bolivianum*. **4–7** male (MZUSP 26083) **4–5** right male palpal bulb **4** retrolateral view **5** prolateral view **6–7** right leg I tibial apophysis (mirrored) **6** prolateral view **7** ventral view **8** female (MHNNKM unnumbered), spermathecae, dorsal view. Scale bar: 1 mm.

**Figures 9–13.**
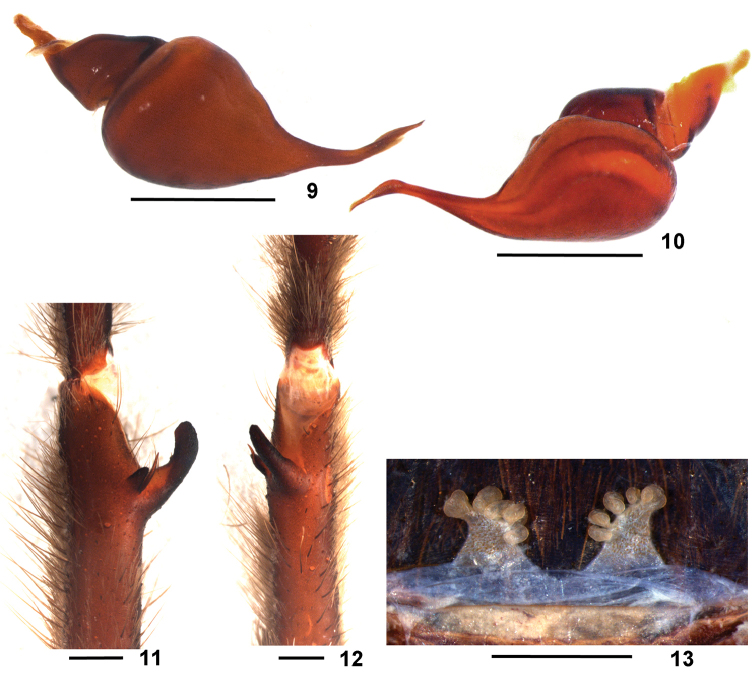
*Dolichothele mottai* sp. n. **9–12** paratype male (DZUB 8248) **9–10**) right male palpal bulb **9** retrolateral view **10** prolateral view **11–12** left leg I tibial apophysis **11** prolateral view **12** ventral view **13** holotype female, spermathecae, dorsal view. Scale bar: 1 mm.

**Figures 14–18.**
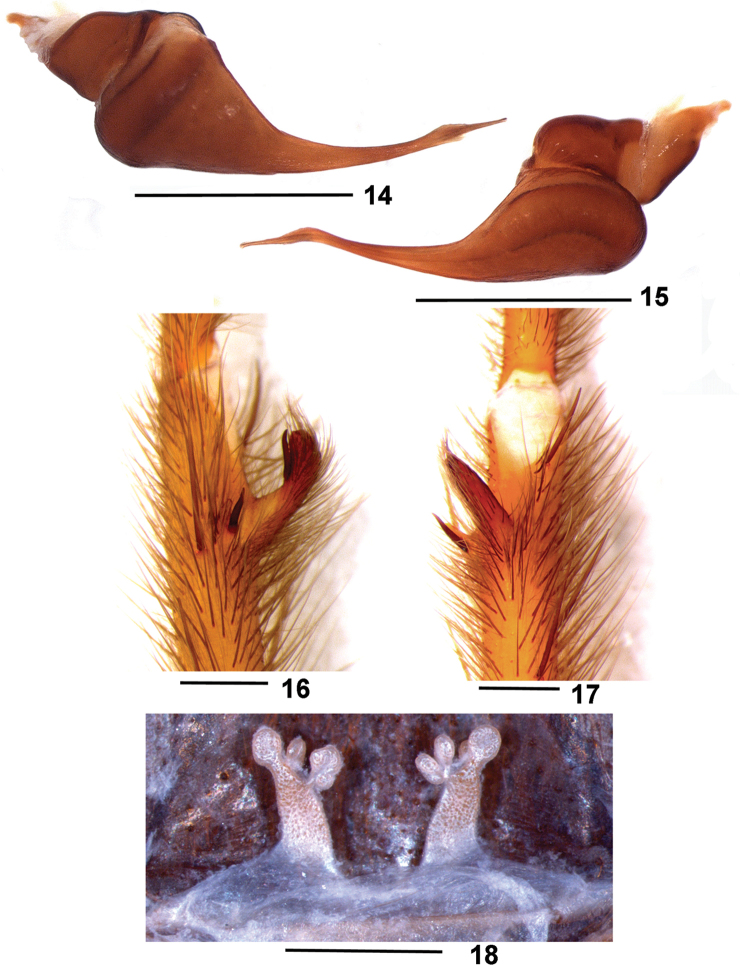
*Dolichothele camargorum* sp. n. **14–17** holotype male (DZUB 8249) **14–15** right male palpal bulb **14** retrolateral view **15** prolateral view **16–17** left leg I tibial apophysis **16** prolateral view **17** ventral view **18** paratype female (DZUB 8250), spermathecae, dorsal view. Scale bar: 1 mm.

**Figures 19–23.**
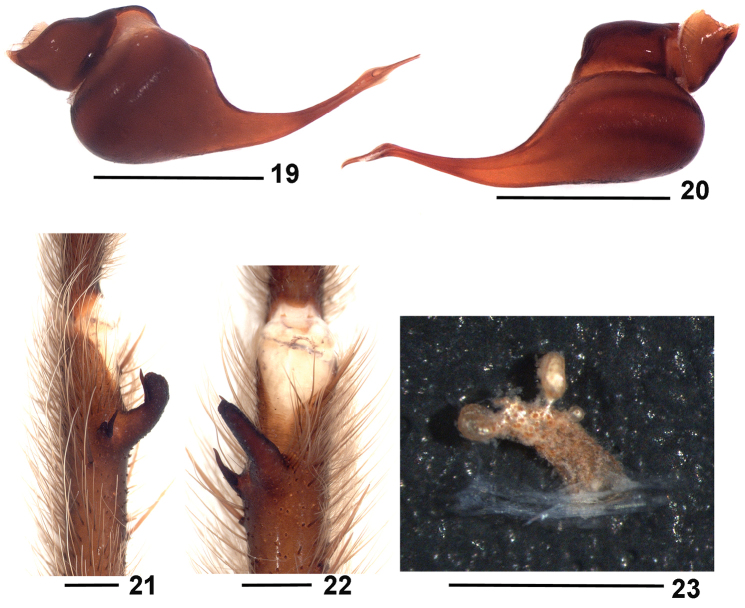
*Dolichothele camargorum* sp. n. **19–22** male (MZUSP 26085) **19–20** right male palpal bulb **19** retrolateral view **20** prolateral view **21–22** right leg I tibial apophysis (mirrored) **21** prolateral view **22** ventral view **23** female (MZUSP 26084), spermathecae, dorsal view. Scale bar: 1 mm.

**Figures 24–27.**
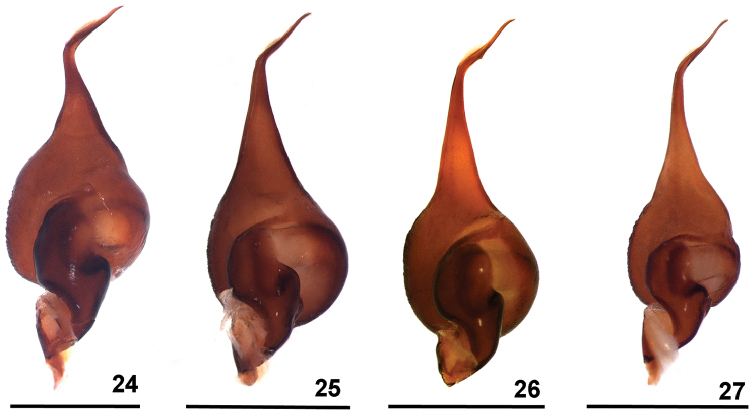
*Dolichothele* spp. Male palpal bulbs, dorsal view. **24** *Dolichothele mottai* sp. n., paratype (DZUB 8248) **25** *Dolichothele bolivianum* (MZUSP 26083) **26** *Dolichothele camargorum* sp. n. (MZUSP 26085) **27** *Dolichothele camargorum* sp. n., holotype (DZUB 8249). Scale bar: 1 mm.

Males of *D. bolivianum* resemble those of *D. dominguense* , *D. camargorum* sp. n., and *D. mottai* sp. n. by the presence of a small subapical keel on male palpal bulb embolus (Figs 1–2, 4–5, 25). They differ from *D. dominguense* (Guadanucci 2007, f. 26–28) by the short embolus; from *D. mottai* sp. n. (Figs 9–10, 24) by the less curved and longer embolus; and from *D. camargorum* sp. n. (Figs 14–15, 19–20, 26–27) by stouter embolus, mainly on its more basal portion. Females of *D. bolivianum* (Fig. 8) resemble those of *D. camargorum* sp. n. (Fig. 18, 23) and *D. mottai* sp. n. (Fig. 13) by the short spermathecae. They differ from *D. camargorum* sp. n. by the shorter and somewhat triangular shape; from *D. mottai* sp. n. they differ by slender spermathecae and cephalothorax covered by brown setae.

#### Redescription.

Holotype (Figs 1–3). Carapace 7.3 long, 5.4 wide, chelicerae 2.6. Legs (femur, patella, tibia, metatarsus, tarsus, total): I: 7.2, 3.9, 5.3, 5.1, 3.6, 25.1. II: 6.5, 3.2, 4.5, 4.2, 3.1, 21.5. III: 5.2, 2.4, 3.6, 4.5, 3.0, 18.7. IV: 7.1, 3.0, 5.6, 6.3, 3.6, 25.7. Palp: 3.9, 2.6, 3.5, – , 1.5, 11.5. Mid-widths: femora I–IV = 1.6, 1.3, 1.6, 1.2, palp = 1.0; patellae I–IV = 1.2, 1.4, 1.0, 1.2, palp = 1.2; tibiae I–IV = 1.1, 1.0, 1.3, 0.9, palp = 1.0; metatarsi I–IV = 1.3, 0.9, 0.7, 0.7; tarsi I–IV = 0.7, 0.7, 0.7, 0.9, palp = 0.9. Abdomen 7.7 long, 3.9 wide. Spinnerets: PMS, 0.7 long, 0.4 wide, 0.3 apart; PLS, 1.4 basal, 1.1 middle, 1.4 distal; mid-widths 0.8, 0.7, 0.6, respectively. Carapace. Length to width 1.35. Fovea: straight, deep, 0.8 wide. Eyes and eye tubercle. Tubercle 0.8 long, 1.1 wide. Clypeus 0.3 wide. Anterior eye row slightly procurved, posterior slightly recurved. Sizes and inter-distances: AME 0.5, ALE 1.1, PME 0.6, PLE 1.0, AME–AME 0.1, AME–ALE 0.1, AME–PME 0.1, ALE–ALE 0.4, ALE–PME 0.2, PME–PME 0.6, PME–PLE 0.1, PLE–PLE 0.7, ALE–PLE 0.2, AME–PLE 0.2. Maxillae: 1.7 long, 1.5 wide, with 20 cuspules spread over ventral inner heel. Labium: 0.3 long, 1.0 wide, with 3 cuspules. Labio-sternal groove shallow, narrow, with two sigilla. Chelicerae: rastelum absent, basal segment with 8 teeth decreasing in size from distal to basal portion. Sternum: 3.7 long, 2.8 wide. Sigilla: three pairs ovals, hardly visible, less than one diameter from margin. Legs: leg formula: IV I II III. Clavate trichobothria: on distal half of tarsi I–IV. Scopula: tarsi I–IV fully scopulate; IV with two rows of setae, not separating the scopula. Metatarsi I–II fully scopulate; III 2/3, IV 1/3 distal scopulate, with two rows of setae, not separating the scopula. Spination: palp: femur p0-0-1, patella v0-2-1, tibia v0-1-1; leg I: femur 0, patella 0, tibia v2-2-2, r2-2-1, metatarsus v0-1-0, p1-1-0, r1-0-0; leg II: femur p0-0-1, patella 0, tibia v1-2-1, p1-1-0; metatarsus v0-1-0; leg III: femur d0-1-0, patella 0, tibia v2-4-1, p1-1-0, r1-1-0, metatarsus v0-2-1ap, p1-1-0, r0-1-2(1ap), d0-0-1ap; IV: femur d0-2-0, patella 0, tibia v2-3-2, p1-1-1, r1-1-0, metatarsus v0-1-1ap, p0-1-1ap, r1-2-1ap. Claws: ITC absent from all legs; STC without teeth. Palpal bulb (Figs 1–2, compare 4–5, and 25 from MZUSP 26083): pyriform, embolus broad at its base, tapering and curved 45° to the retrolateral side on its distal third, with a small keel just after the curvature. Embolus the same or a little longer than tegulum. Male tibial apophysis (Fig. 3, compare Figs 6–7) with two branches originating from a common low base, positioned distant from metatarsus. Retrolateral branch longer than prolateral, not dilated on distal portion, with a spine on its mid-length. Prolateral branch shorter than contiguous spine. Both branches inclined ca. 45° to the prolateral side. Metatarsus I slightly curved. Color pattern. Carapace, chelicerae and legs dorsally brown, covered with light brown setae. Distal metatarsi and tarsi darker. Carapace with long light brown setae. Sternum, labium, maxillae and coxae light brown. Other leg articles ventrally brown. Abdomen dark brown covered with short golden setae and long light brown setae. Distal femora, patellae, tibiae and metatarsi with narrow whitish rings. Longitudinal stripes on leg articles not evident.

#### Female

MHNNKM unnumbered. Carapace 7.8 long, 6.1 wide, chelicerae 3.1. Legs (femur, patella, tibia, metatarsus, tarsus, total): I: 5.3, 3.3, 3.5, 3.1, 2.3, 19.8. II: 4.9, 3.3, 3.1, 3.0, 2.2, 16.5. III: 4.2, 2.8, 2.6, 3.0, 2.1, 14.7. IV: 5.6, 3.6, 4.5, 4.6, 2.5, 20.8. Palp: 4.2, 2.5, 2.3, – , 2.4, 11.4. Mid-widths: femora I–IV = 1.4, 1.5, 1.5, 1.2, palp = 1.2; patellae I–IV = 1.2, 1.3, 1.4, 1.3, palp = 1.2; tibiae I–IV = 1.3, 1.3, 1.1, 1.3, palp = 1.3; metatarsi I–IV = 1.0, 1.0, 1.1, 0.8; tarsi I–IV = 1.3, 1.2, 1.1, 1.1, palp = 1.3. Abdomen 10.1 long, 6.4 wide. Spinnerets: PMS 0.92 long, 0.42 wide, 0.33 apart; PLS 1.42 basal, 1.02 middle, 1.85 distal; mid-widths 0.98, 0.84, 0.70, respectively. Carapace: length to width 1.27. Fovea: straight, deep, 0.66 wide. Eyes and eye tubercle. Tubercle 0.97 long, 1.36 wide. Clypeus 0.10 wide. Anterior eye row slightly procurved, posterior eye row slightly recurved. Sizes and inter-distances: AME 0.42, ALE 0.41, PME 0.22, PLE 0.28, AME–AME 0.07, AME–ALE 0.08, AME–PME 0.06, ALE–ALE 0.75, ALE–PME 0.30, PME–PME 0.66, PME–PLE 0.09, PLE–PLE 1.05, ALE–PLE 0.16, AME–PLE 0.30. Eye group 1.36 wide, 0.72 long. Maxillae: 2.22 long, 1.35 wide, with 18 cuspules spread over ventral inner heel. Lyra absent. Labium: 0.61 long, 1.20 wide, with 4 cuspules. Labio-sternal groove shallow, narrow, with two sigilla. Chelicerae: rastellum absent, basal segment with 8 teeth decreasing in size from distal to basal portion, with small teeth on basal area. Sternum: 3.64 long, 3.12 wide. Posterior angle rounded, not separating coxae IV. Sigilla: three pairs, all small, rounded, hardly visible, less than one diameter from margin. Legs: leg formula: IV I II III. Clavate trichobothria: on distal 2/3 of tarsi I–IV. Scopula: tarsi I–IV fully scopulate, IV with two rows of setae, not separating the scopula. Metatarsi I–II fully scopulate; III–IV 2/3 distal scopulate, IV with two rows of setae, not separating the scopula. Spination: palp: femur 0, patella 0, tibia v0-0-1ap; leg I: femur 0, patella 0, tibia v0-0-1ap, metatarsus v1-0-0; leg II: femur p0-0-1, patella 0, tibia 0; metatarsus v1-0-0; leg III: femur 0, patella 0, tibia v0-0-2ap, p0-1-0, r1-1-0, metatarsus v2-0-2ap, p1-2-2(1ap), r0-1-1; IV: femur r0-0-1, patella 0, tibia v0-1-2ap, r1-0-1, metatarsus v2-0-1ap, p0-1-2(1ap), r1-0-3(2ap). Claws: STC lacking teeth. Genitalia (Fig. 8): Spermathecae short, triangular, with 5–6 lobes on internal side, from tip to base. Color pattern (Fig. 31): as in male.

Immatures (Fig. 29) have black carapace and abdomen and reddish brown legs, except for the black tarsi.

#### Distribution.

Bolivia, department of Santa Cruz; and Brazil, state of Mato Grosso (Fig. 36).

#### Remarks.

Guadanucci (2007) examined only males from the type locality of *D. bolivianum*, even though a photo of a female (Guadanucci 2007, f. 9) is shown in his paper. The female of *D. bolivianum* was originally described based on a casting skin by Vol (2001), and the specimen, a paratype, was not located in the MHNNKM where it should be deposited. Thus, herein, the topotypical female is described and illustrated for first time.

#### Ecology.

**Figures 28–35.**
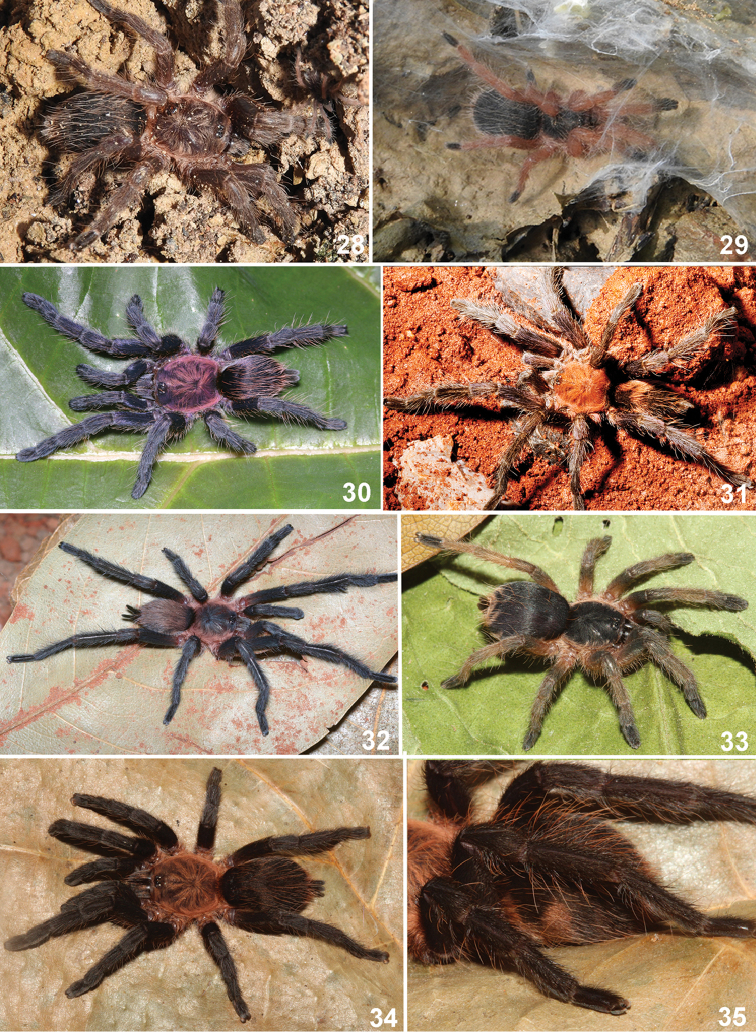
*Dolichothele* species. **28–29** *Dolichothele bolivianum* **28** female **29** immature. Both from Bolivia, Santa Cruz, Samaipata **30–31** *Dolichothele mottai* sp. n. **30** female **31** male. Both from Brazil, Distrito Federal, Brasília **32–35** *Dolichothele camargorum* sp. n. from Brazil, Rondônia, Monte Negro **32** male **33** immature **34** female (DZUB 8250) **35** female (DZUB 8250), detail of lateral abdomen showing stripes. Photographs IS Revollo and RB Huanto (**28–29**). PC Motta (**31**),R Bertani (**30, 32–35**).

The species is found in Bosque montañoso (Bolivian Montaine Dry Forests) in Bolivia and Cerrado (a type of savannah vegetation) in Brazil. One of the authors (ISR) collected females and immatures under rocks on the way to mountains on Samaipata (Figs 28–29), Santa Cruz, Bolivia, during the day and afternoon (December 2015).

### Dolichothele mottai sp. n.

Taxon classificationAnimaliaAraneaeTheraphosidae

http://zoobank.org/2D8F4B25-9226-44FD-BEA8-B1CADF52BD27

Figs 9–13 , 24 , 30–31 , 36

Oligoxystre bolivianum ; Guadanucci 2007: 4, f. 1–12 (in part, only f. 1–8).
Dolichothele bolivianum ; Lucas and Indicatti 2015: 207 (in part).

#### Type material.

Holotype female, BRAZIL: *Distrito Federal*, Brasília, Reserva Ecológica do IBGE [16°56'S; 47°53'W], 10 July 2007, R. Bertani, P. Motta, C. S. Fukushima, R. H. Nagahama, J. Crisóstomo coll. (DZUB 8246); paratype male, BRAZIL: *Distrito Federal*, Brasília [15°47'S; 47°52'W], without additional data (DZUB 8248).

#### Other material.

**BRAZIL**: *Distrito Federal*, Brasilia, 1 male, without additional data (DZUB 131); SHIS – QI 26 Chac. 17 [15°49'S; 47°48'W], 1 female, 29 September 2002, S. S. Salles coll. (DZUB 343); Reserva Ecológica do IBGE [16°56'S; 47°53'W], cerrado, termite mound, 1 male, 02 September 2002, J. R. R. Pinto coll. (DZUB 1129); IBGE, cerrado, 43JC, 1 male, 02 October 2003, M. Milhomem coll. (DZUB 870); IBGE, termite mound, 1 female, 02 October 2014, R. Japiassu coll. (DZUB 6741); Centro de Instrução e Adestramento de Brasília-CIAB-Marinha (16°00’6.73”S; 47°57’5.82”W), termite mound, 1 female, 11 July 2007, R. Bertani, P. C. Motta, C. S. Fukushima, R. H. Nagahama, J. Crisóstomo coll. (DZUB 8247); Sobradinho [15°39'S; 47°47'W], Cond. Fraternidade, DF425, casa, 1 male, 06 September 2006, P. C. Motta coll. (DZUB 1824); same data, 1 female, 06 November 2006 (DZUB 1992); same data, 1 female, 11 January 2007, P. C. Motta coll. (DZUB 2099); Córrego do Urubú, 1 male, 06 October 2009, I. Wagas coll. (DZUB 3951), 1 male, 29 October 2007, J. Marinho coll. (DZUB 2752); *Goiás*: Aragarças [16°05'S; 52°14'W], 1 male, 15 July 1976, L. Edmundo coll. (MNRJ 03850); Caldas Novas [17°44'S; 48°37'W], P. E. Pescan, Cerrado s. s., coleta ativa diurna, 1 male, 01 November 2014, P. C. Motta *et al.* coll. (DZUB 7592); Catalão [18°09'S; 47°56'W] (Fazenda Alvorada), 1 male, January 2004, J. P. L. Guadanucci & A. Monteiro coll. (MZUSP 26076), 1 female, February 2003, J. P. L. Guadanucci coll. (MZUSP 23224); Cocalzinho de Goiás [15°46'S; 48°46'W], 1 male, 07 October 2011, I. R. Pereira Silva coll. (DZUB 4788); Mineiros, Parque Nacional das Emas [18°08’S; 52°55’W], 1 male, 5 September 1997, C. Nogueira & P. Valdujo coll. (IBSP 109493).

#### Differential diagnosis.

Males of *D. mottai* sp. n. resemble those of *D. dominguense* (Guadanucci 2007, f. 26–28), *D. camargorum* sp. n. (Figs 14–15, 19–20, 26–27) and *D. bolivianum* (Figs 1–2, 4–5, 25) by the presence of a small subapical keel on male palpal bulb embolus. They differ from all these species by the very short and strongly curved embolus (Figs 9–10, 24). Females of *D. mottai* sp. n. (Fig. 13) resemble those of *D. camargorum* sp. n. (Fig. 18, 23) and *D. bolivianum* (Fig. 8) by the short spermathecae. They differ from *D. camargorum* sp. n. by the shorter and somewhat triangular spermathecae shape; from *D. bolivianum* sp. n. they differ by broader spermathecae. Additionally, males and females differ from all *Dolichothele* species by the carapace covered with iridescent red setae (Figs 28–29).

#### Description.

Female holotype (DZUB 8246). Carapace 9.4 long, 8.3 wide, chelicerae 4.3. Legs (femur, patella, tibia, metatarsus, tarsus, total): I: 7.1, 4.7, 4.4, 4.5, 2.9, 23.6. II: 6.6, 4.4, 4.3, 4.2, 2.5, 22.0. III: 6.0, 3.5, 3.7, 4.4, 2.7, 20.3. IV: 7.1, 4.2, 5.4, 6.1, 2.9, 25.7. Palp: 5.4, 3.6, 3.2, –, 2.9, 15.1. Mid-widths: femora I–IV = 1.5, 1.5, 1.6, 1.7, palp = 1.3; patella I–IV = 1.1, 1.2, 1.2, 1.4, palp = 1.3; tibiae I–IV = 1.5, 1.3, 1.2, 1.2, palp = 1.3; metatarsi I–IV = 1.2, 1.3, 1.2, 0.9; tarsi I–IV = 1.3, 1.3, 1.1, 1.0, palp = 1.5. Abdomen 10.8 long, 5.3 wide. Spinnerets: PMS, 1.36 long, 0.63 wide, 0.41 apart; PLS, 2.27 basal, 1.24 middle, 1.48 distal; mid-widths: 1.11, 1.08, 0.81, respectively. Carapace: length to width 1.13. Fovea: straight, deep, 1.30 wide. Eyes and eye tubercle. Tubercle 1.07 long, 1.39 wide. Clypeus 0.20 wide. Anterior row slightly procurved, posterior row slightly recurved. Sizes and inter-distances: AME 0.37, ALE 0.35, PME 0.28, PLE 0.30, AME–AME 0.36, AME–ALE 0.15, AME–PME 0.08, ALE-ALE 1.00, ALE–PME 0.30, PME–PME 0.86, PME–PLE 0.08, PLE–PLE 1.22, ALE–PLE 0.22, AME–PLE 0.30. Eye group 1.39 wide, 0.75 long. Maxillae: 3.08 long, 1.41 wide, with 22 cuspules spread over ventral inner heel. Lyra absent. Labium: 0.62 long, 1.38 wide, with 4 cuspules. Labio-sternal groove shallow, narrow, with two sigilla. Chelicerae: rastellum absent, basal segment with 8 teeth decreasing in size from distal to basal portion; and small teeth on basal area. Sternum: 4.53 long, 4.26 wide. Posterior angle rounded, not separating coxae IV. Sigilla: three pairs, all small, rounded, less than one diameter from margin. Legs: leg formula: IV I II III. Clavate trichobothria: on distal 2/3 of tarsi I–IV. Scopula: Tarsi I–IV fully scopulate, IV with two rows of setae, not separating the scopula. Metatarsi I–III fully scopulate; IV 2/3 scopulate, with two rows of setae, not separating the scopula. Spination: palp: femur p0-0-2, patella 0, tibia v0-2-2(1ap), metatarsus 0; leg I: femur p0-0-1, patella 0, tibia v0-1-1ap, p0-0-1, metatarsus v1-0-0; leg II: femur p0-0-1, patella 0, tibia v0-1-1ap, p1-0-1, metatarsus v1-0-0; leg III: femur 0, patella 0, tibia v0-2-2ap, p1-0-1, r0-0-1; metatarsus v1-1-2ap, p1-0-1ap, r0-1-0; IV: femur r0-0-1, patella 0, tibia v1-2-3(2ap), p0-0-1, r0-0-1, metatarsus v2-0-2ap, p1-1-1, r0-1-1. Claws: ITC absent from all legs; STC lacking teeth. Genitalia (Fig. 13). Spermathecae short, triangular, with ca. 6 lobes on internal side, from tip to base. Color pattern (Fig. 28). Carapace brown covered with long metallic reddish setae. Chelicerae and legs ventrally and dorsally dark, except for black femora. Sternum, labium, maxillae, and coxae brown. Abdomen ventrally brown, dorsally black. Distal femora, patellae, tibiae and metatarsi rings not evident. Longitudinal stripes on leg articles not evident.

#### Male paratype

(DZUB 8248). Carapace 8.1 long, 7.3 wide, chelicerae 3.5. Legs (femur, patella, tibia, metatarsus, tarsus, total): I: 8.3, 4.6, 5.6, 5.5, 3.6, 27.6. II: 7.2, 4.4, 4.0, 5.1, 3.2, 23.9. III: 6.2, 3.3, 4.1, 5.2, 3.3, 22.1. IV: 8.2, 4.0, 6.4, 7.3, 3.7, 29.6. Palp: 4.9, 3.3, 3.7, – , 1.6, 13.5. Mid-widths: femur I–IV = 1.9, 1.8, 1.7, 1.7, palp = 1.5; patella I–IV = 1.5, 1.6, 1.6, 1.5, palp = 1.4; tibiae I–IV = 1.2, 1.3, 1.2, 1.2, palp = 1.3; metatarsi I–IV = 0.9, 0.9, 0.9, 0.8; tarsi I–IV = 1.1, 1.0, 1.0, 1.0, palp = 1.2. Abdomen 8.8 long, 5.0 wide. Spinnerets: PMS, 0.76 long, 0.45 wide, 0.25 apart; PLS, 1.13 basal, 1.06 middle, 1.60 distal; mid-widths: 0.51, 0.53, 0.39, respectively. Carapace. Length to width 1.10. Fovea: straight, deep, 1.37 wide. Eyes and eye tubercle. Tubercle 1.12 long, 1.51 wide. Clypeus 0.09 wide. Anterior eye row slightly procurved, posterior eye row slightly recurved. Sizes and inter-distances: AME 0.41, ALE 0.38, PME 0.26, PLE 0.38, AME–AME 0.08, AME–ALE 0.10, AME–PME 0.02, ALE–ALE 0.85, ALE–PME 0.29, PME–PME 0.74, PME–PLE 0.08, PLE–PLE 1.17, ALE–PLE 0.18, AME–PLE 0.31. Eye group 1.51 wide, 0.79 long. Maxillae: 2.52 long, 1.35 wide, with 8 cuspules spread over ventral inner heel. Lyra absent. Labium: 0.48 long, 1.32 wide, with 2 cuspules. Labio-sternal groove shallow, narrow, with two sigilla. Chelicerae: rastellum absent, basal segment with 8 teeth decreasing in size from distal to basal portion, with very small denticles on base. Sternum: 4.41 long, 2.70 wide. Posterior angle rounded, not separating coxae IV. Sigilla: three pairs, all small, rounded, less than one diameter from margin. Legs: leg formula: IV I II III. Clavate trichobothria: on distal 2/3 of tarsi I–IV. Scopula: tarsi I–IV fully scopulate, IV with two rows of setae, not separating the scopula. Metatarsi I–II fully scopulate; III–IV 2/3 scopulate. IV with two rows of setae, not separating the scopula. Spination: palp: femur p0-0-2, patella 0, tibia 0; leg I: femur p0-0-1, patella 0, tibia v0-1-0, p0-1-0, metatarsus 0; leg II: femur p0-0-1; patella 0, tibia v0-2-1ap, p1-0-1, metatarsus v1-0-0; leg III: femur 0, patella 0, tibia v0-2-2ap, p1-1-0, r1-0-1, metatarsus v1-0-0, r0-1-1; leg IV: femur r0-0-1, patella 0, tibia v0-1-0, p1-0-0, r2-0-1, metatarsus v1-0-1ap, p1-1-1, r0-1-1. Claws: ITC absent from all legs; STC lacking teeth. Palpal bulb (Figs 9–10, 24): pyriform, embolus narrowing abruptly at its base and curved slightly to prolateral and then 45° to the retrolateral side on its distal third (“s” shape, as seen from above), with a small keel just after the curvature. Embolus shorter than tegulum. Male tibial apophysis (Figs 11–12) with two branches originating from a common low base, positioned distant from metatarsus. Retrolateral branch longer than prolateral, not dilated on distal portion, with a spine on its mid-length. Prolateral branch shorter than contiguous spine. Both branches inclined ca. 45° to the prolateral side. Metatarsus I slightly curved. Color pattern (Fig. 29): as in female, except chelicerae and trochanters dorsally reddish and abdomen with long reddish setae.

#### Etymology.

The specific name is a patronym in honor of the arachnologist Dr. Paulo Cesar Motta, for his contributions to the taxonomy and biology of mygalomorphs inhabiting the Brazilian Cerrado region.

#### Distribution.

**Figure 36.**
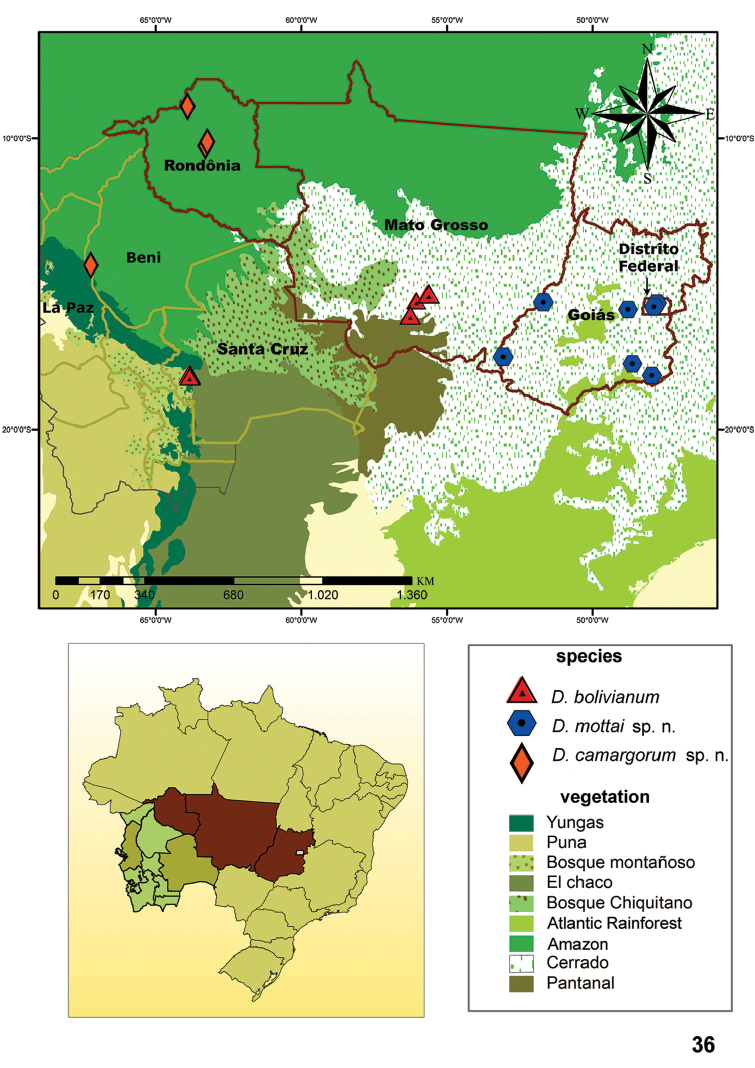
Map showing records of *D. bolivianum*, *D. mottai* sp. n., and *D. camargorum* sp. n.

Brazil, Distrito Federal and state of Goiás (Fig. 36).

#### Remarks.

The specimens used by Guadanucci (2007) to redescribe *D. bolivianum* (MZUSP 26076 and MZUSP 23224) were reanalyzed and belong to *D. mottai* sp. n. The female specimen (IBSP 103094) from Miranda (Agachi), state of Mato Grosso do Sul, Brazil, cited by Lucas and Indicatti (2015) as *D. bolivianum* has long and slender spermathecae with several lobes on apex and laterals. Therefore, it seems related with forms from eastern Brazil (see Guadanucci 2007, 2011), and probably the locality is a label mistake.

#### Ecology.

*Dolichothele mottai* sp. n. occurs on the Cerrado *stricto* *sensu* from Central-Western Brazil. The female constructs silk tunnels under rocks and logs, and males were found moving between September and November when they leave their shelter to search for females, in Distrito Federal (Motta 2014).

### Dolichothele camargorum sp. n.

Taxon classificationAnimaliaAraneaeTheraphosidae

http://zoobank.org/FB361627-5A32-4722-BB96-92ADB1A8D78D

Figs 14–18 , 19–23 , 26–27 , 32–35 , 36

Oligoxystre bolivianum ; Guadanucci 2007: 4, f. 1–12 (in part, f. 10–12).
Dolichothele bolivianum ; Lucas and Indicatti 2015: 207 (in part).

#### Type material.

Male holotype (DZUB 8249). BRAZIL: *Rondônia*: Monte Negro [10°15'S; 63°17'W], nighttime hand collecting, 23 July 2007, P. I. Silva Jr., R. Bertani & R. Martins coll. (DZUB 8249); female paratype (DZUB 8250), BRAZIL: *Rondônia*: Monte Negro [10°15'S; 63°17'W] BR421, km 30, 20 December 2013, P. I. Silva Jr coll.

#### Other material.

**BRAZIL**, *Rondônia*: Monte Negro [10°15'S; 63°17'W], BR421, km 30, daytime hand collecting, 1 female, 18 December 2013, P. I. Silva Jr coll. (DZUB 8251); 1 female, 18 December 2013, P. H. Martins *et al.* coll. (UFMG 17214); Porto Velho, Mutum [8°33'S; 63°42'W], 1 male, 18 April 2012, R. P. Indicatti coll. (MZUSP 51008); BOLÍVIA, *La Paz*: San Buenaventura [14°27'S; 67°35'W], 1 female, 04 October 2004, D. Weinmann & A. Stirm coll. (MZUSP 26084); 1 male, 04 October 2004, D. Weinmann & A. Stirm coll. (MZUSP 26085).

#### Differential diagnosis.

Males of *D. camargorum* sp. n. (Figs 14–15, 19–20, 27) resemble those of *D. dominguense* (Guadanucci 2007, f. 26–28), *D. bolivianum* sp. n. (Figs 1–2, 4–5, 25) and *D. mottai* sp. n. (Figs 9–10, 24) by the presence of a small subapical keel on male palpal bulb embolus. They differ from *D. dominguense* by the short embolus; from *D. mottai* sp. n. by the less curved and longer embolus; and from *D. bolivianum* by the slender embolus. Females of *D. camargorum* sp. n. (Figs 18, 23) resemble those of *D. bolivianum* and *D. mottai* sp. n. by the short spermathecae. They differ from both *D. bolivianum* and *D. mottai* sp. n. by the long and narrow spermathecae shape.

#### Description.

Male holotype (DZUB 8249). Carapace 5.6 long, 5.2 wide, chelicerae 2.5. Legs (femur, patella, tibia, metatarsus, tarsus, total): I: 6.2, 3.4, 5.1, 5.0, 3.6, 23.3 II: 6.5, 3.1, 4.3, 4.7, 3.0, 21.6. III: 5.2, 2.2, 3.6, 4.1, 3.0, 18.1. IV: 6.6, 3.0, 5.1, 6.5, 3.4, 24.6. Palp: 4.2, 2.1, 3.5, – , 1.3, 11.1. Mid-widths: femur I–IV = 1.0, 1.1, 1.2, 0.9, palp = 1.0; patella I–IV = 1.0, 1.1, 1.0, 0.6, palp = 0.9; tibiae I–IV = 1.0, 0.5, 0.7, 0.7 palp = 1.0; metatarsi I–IV = 0.9, 0.7, 0.7, 0.7; tarsi I–IV = 0.8, 0.7, 0.8, 0.6, palp = 0.7. Abdomen 6.8 long, 4.9 wide. Spinnerets: PMS, 0.66 long, 0.27 wide, 0.25 apart; PLS, 1.20 basal, 1.16 middle, 1.98 distal; mid-widths: 0.56, 0.52, 0.39, respectively. Carapace: length to width 1.07. Fovea: slightly procurved, deep, 0.86 wide. Eyes and eye tubercle. Tubercle 0.89 long, 1.23 wide. Clypeus 0.05 wide. Anterior row slightly procurved, posterior row slightly recurved. Sizes and inter-distances: AME 0.38, ALE 0.32, PME 0.23, PLE 0.29, AME–AME 0.11, AME–ALE 0.14, AME–PME 0.43, ALE–ALE 0.71, ALE–PME 0.23, PME–PME 0.64, PME–PLE 0.40, PLE–PLE 0.90, ALE–PLE 0.17, AME–PLE 0.27. Eye group 1.23 wide, 0.64 long. Maxillae: 1.96 long, 1.03 wide, with 22 cuspules spread over ventral inner heel. Lyra absent. Labium: 0.41 long, 0.96 wide, with 4 cuspules. Labio-sternal groove shallow, narrow, with two sigilla. Chelicerae: rastellum absent, basal segment with 8 teeth decreasing in size from distal to basal portion, with very small denticles on base. Sternum: 3.07 long, 2.62 wide. Posterior angle rounded, not separating coxae IV. Sigilla: sigilla not evident. Legs: leg formula: IV I II III. Clavate trichobothria: on distal 2/3 of tarsi I–IV. Scopula: tarsi I–IV fully scopulate, IV with sparse setae, not separating the scopula. Metatarsi I fully scopulate; II–IV 2/3 distal scopulate, IV with sparse setae, not separating the scopula. Spination: palp: femur p0-0-1, patella 0, tibia p0-1-0; leg I: femur p0-0-1, patella 0, tibia v0-0-2(1ap), p0-0-2, r2-2-1ap, metatarsus p0-1-0, r1-0-0; leg II: femur p0-1-1, patella 0, tibia v2-2-2(1ap), p1-1-0, metatarsus v1-0-0; leg III: femur p1-1-1, r1-1-1, d1-0-0, patella 0, tibia v2-3-2ap, p1-0-1, r1-0-1, metatarsus v 1-1-1ap, p1-1-2(1ap), r0-1-1; leg IV: femur p0-0-1, r0-0-1, patella 0, tibia v3-2-1ap, p 1-0-1, r 0-1-0, metatarsus v2-0-2ap, p1-1-1, r1-1-1. Claws: ITC absent from all legs; STC lacking teeth. Palpal bulb (Figs 14–15, 27, compare 19–20, and 26 from MZUSP 26085): pyriform, embolus narrowing abruptly at its base and curved 45° to the retrolateral side on its distal third, with a small keel just after the curvature. Embolus longer than tegulum. Male tibial apophysis (Figs 16–17, compare 21–22) with two branches originating from a common low base, positioned distant from metatarsus. Retrolateral branch longer than prolateral, not dilated on distal portion, with a spine on its mid-length. Prolateral branch shorter than contiguous spine. Both branches inclined ca. 45° to the prolateral side. Metatarsus I slightly curved. Color pattern (Fig. 32). Carapace black bordered by light brown long setae. Chelicerae and legs dorsally and ventrally black. Sternum, labium, maxillae, and coxae brown. Abdomen ventrally brown, dorsally black. Distal femora, patellae, tibiae and metatarsi rings not evident. Longitudinal stripes on leg articles not evident.

#### Female paratype

(DZUB 8250). Carapace 10.9 long, 8.2 wide, chelicerae 5.5. Legs (femur, patella, tibia, metatarsus, tarsus, total): I: 8.0, 5.3, 5.7, 4.9, 3.3, 27.2. II: 7.4, 5.0, 4.9, 5.1, 3.8, 26.5. III: 7.2, 3.9, 4.7, 5.5, 3.7, 25. IV: 9.0, 4.7, 7.0, 7.7, 3.8, 32.2. Palp: 6.0, 3.8, 3.9, – , 4.7, 18.4. Mid-widths: femora I–IV =2.1, 1.7, 1.5, 1.7, palp = 1.6; patella I–IV = 1.9, 1.8, 1.7, 1.5, palp = 1.6; tibiae I–IV = 1.7, 1.2, 1.4, 1.5, palp = 1.5; metatarsi I–IV = 1.5, 1.3, 1.2, 1.1; tarsi I–IV = 1.2, 1.1, 1.2, 1.3, palp = 1.6. Abdomen 12.2 long, 7.6 wide. Spinnerets: PMS, 1.17 long, 0.62 wide, 0.30 apart; PLS, 2.33 basal, 1.81 middle, 2.70 distal; mid-widths: 1.27, 1.22, 0.93, respectively. Carapace: length to width 1.32. Fovea: slightly procurved, deep, 1.24 wide. Eyes and eye tubercle. Tubercle 1.30 long, 1.83 wide. Clypeus 0.10 wide. Anterior row slightly procurved, posterior row slightly recurved. Sizes and inter-distances: AME 0.46, ALE 0.49, PME 0.29, PLE 0.42, AME–AME 0.12, AME–ALE 0.15, AME–PME 0.07, ALE–ALE 1.05, ALE–PME 0.26, PME–PME 1.02, PME–PLE 0.12, PLE–PLE 1.37, ALE–PLE 0.22, AME–PLE 0.38. Eye group 1.83 wide, 0.96 long. Maxillae: 3.67 long, 1.86 wide, with 16 cuspules spread over ventral inner heel. Lyra absent. Labium: 0.71 long, 1.68 wide, with 2 cuspules. Labio-sternal groove shallow, narrow, with two sigilla. Chelicerae: rastellum absent, basal segment with 9 teeth decreasing in size from distal to basal portion, and small teeth on basal area. Sternum: 5.21 long, 4.17 wide. Posterior angle rounded, not separating coxae IV. Sigilla: three pairs, all small, rounded, less than one diameter from margin. Legs: leg formula: IV I II III. Clavate trichobothria: on distal 2/3 of tarsi I-IV. Scopula: tarsi I–IV fully scopulate, IV with two rows of setae, not separating the scopula. Metatarsi I–II fully scopulate; III–IV 3/4 distal scopulate with two rows of setae, not separating the scopula. Spination: palp: femur p0-0-1, patella 0, tibia v0-2-3(2ap), p0-1-0; leg I: femur 0, patella 0, tibia v0-1-1ap, p0-0-1, metatarsus v1-0-0; leg II: femur p0-0-1, patella 0, tibia v0-1-1ap, p0-0-1; metatarsus v1-0-0; leg III: femur p0-1-1, r0-1-1, patella 0, tibia v1-2-2ap, p0-1-1, r 0-1-1, metatarsus v2-0-3ap, p1-1-1, r0-1-1; IV: femur r0-0-1, patella 0, tibia v1-2-2, p0-1-0, r1-0-1, metatarsus v2-0-3ap, p0-1-1, r1-0-1. Claws: ITC absent from all legs; STC lacking teeth. Genitalia: Spermathecae (Fig. 18, compare 23) short, longer than wide, rectangular, with 4 lobes on its tip. Color pattern (Figs 34–35). Carapace brown covered with long light brown setae. Chelicerae dark brown. Legs dorsally brown, covered with dark brown setae. Sternum, labium, maxillae, and coxae light brown. Other leg articles ventrally brown. Abdomen ventrally brown, dorsally dark brown extending laterally and forming four wide marks (Fig. 35). Distal femora, patellae, tibiae and metatarsi with narrow whitish rings. Longitudinal stripes on leg articles not evident.

Immatures (Fig. 33) have black carapace and abdomen and the legs are dorsally greyish to brownish with black tarsi. The abdomen dorsum shows broad black marks on the laterals and a narrow posterior black stripe (Fig. 33). Adult females have only four broad black marks extending laterally.

#### Etymology.

The specific name is a patronym in honor of Dr. Erney F. Plessmann de Camargo and Dr. Luis Marcelo Aranha Camargo for their efforts to develop medical and biological research in the state of Rondônia, Brazil. They encouraged the field work on which the specimens of this new species were collected.

**Remarks**. Guadanucci (2007) examined a single specimen of this new species from Brazil, a female from State of Rondônia, Porto Velho, U. H. Samuel (IBSP 9506). This specimen was not examined here, as it was destroyed by a fire in the Instituto Butantan collection buildings in 2010. Other specimens examined from nearby localities show the female has very distinct spermathecae, slender and with lobes only on their tips (Figs 18, 23). The male has a more slender embolus, mainly on its base (Figs 14–15, 27). Males, females and immatures have distinct color patterns from those of *D. bolivianum* and *D. mottai* sp. n. Male and female from Rurrenabaque, Beni, Bolivia shown in Guadanucci (2007) f. 11–12 have a distinct color pattern. As only two males and a female were examined from Bolivia, it is not possible to conclude whether it is morphological variation or another undescribed species. For this reason, figures of male palpal bulb and spermathecae were included to show the morphological variation in the specimens from the two distant localities (Figs 19–21, 26).

#### Distribution.

Brazil, state of Rondônia; and Bolivia, department of La Paz (Fig. 36).

#### Ecology.

*Dolichothele camargorum* sp. n. occurs in the Amazon region, probably in Cerrado remnants.

#### Discussion.

Guadanucci (2007, 2011) recognized eight species in *Oligoxystre* Vellard, 1924 (now *Dolichothele*). One of these species, *D. bolivianum*, was considered to have a wide distribution from Central-Western Brazil to Bolivia, close to the Andes (Guadanucci 2007). Guadanucci (2007) found variation in color pattern throughout the distribution of this species but considered them as local population variation. Examining the available material of Guadanucci (2007) together with additional specimens recently collected, it is possible to recognize two more species, which are herein described. *Dolichothele mottai* sp. n. males clearly have a shorter embolus with a strong “S”-shaped curvature (Figs 9–10, 24), distinct from the longer and straighter embolus of *D. bolivianum* (Figs 1–2, 4–5, 25). *Dolichothele mottai* sp. n. females have a broader spermathecae than those of *D. bolivianum* (Fig. 13) and, as the male, have the carapace with a distinct color pattern of iridescent reddish setae covering it (Figs 30–31). Another new species also closely related with *D. bolivianum* was recognized from the state of Rondônia, Brazil and department La Paz, Bolivia. Males of the new species *D. camargorum* sp. n. have a slender embolus (Figs 14–15, 19–20, 27), when comparing with *D. bolivianum*, and the females have narrow spermathecae with lobes restricted to their apex (Fig. 18). The color pattern is also distinct, males have a dark carapace with orange setae on its borders and the females have dark marks on the lateral abdomen (Figs 33, 35), character unknown in other species of *Dolichothele*.

## Supplementary Material

XML Treatment for Dolichothele

XML Treatment for Dolichothele bolivianum

XML Treatment for Dolichothele mottai

XML Treatment for Dolichothele camargorum
